# ID3RSNet: cross-subject driver drowsiness detection from raw single-channel EEG with an interpretable residual shrinkage network

**DOI:** 10.3389/fnins.2024.1508747

**Published:** 2025-01-08

**Authors:** Xiao Feng, Zhongyuan Guo, Sam Kwong

**Affiliations:** ^1^School of Communications and Information Engineering, Chongqing University of Posts and Telecommunications, Chongqing, China; ^2^Henan High-speed Railway Operation and Maintenance Engineering Research Center, Zhengzhou, Henan, China; ^3^College of Electronic and Information Engineering, Southwest University, Chongqing, China; ^4^Department of Computer Science, City University of Hong Kong, Hong Kong SAR, China; ^5^School of Data Science, Lingnan University, Hong Kong SAR, China

**Keywords:** single-channel EEG, drowsiness detection, residual shrinkage network, attention, interpretability

## Abstract

Accurate monitoring of drowsy driving through electroencephalography (EEG) can effectively reduce traffic accidents. Developing a calibration-free drowsiness detection system with single-channel EEG alone is very challenging due to the non-stationarity of EEG signals, the heterogeneity among different individuals, and the relatively parsimonious compared to multi-channel EEG. Although deep learning-based approaches can effectively decode EEG signals, most deep learning models lack interpretability due to their black-box nature. To address these issues, we propose a novel interpretable residual shrinkage network, namely, ID3RSNet, for cross-subject driver drowsiness detection using single-channel EEG signals. First, a base feature extractor is employed to extract the essential features of EEG frequencies; to enhance the discriminative feature learning ability, the residual shrinkage building unit with attention mechanism is adopted to perform adaptive feature recalibration and soft threshold denoising inside the residual network is further applied to achieve automatic feature extraction. In addition, a fully connected layer with weight freezing is utilized to effectively suppress the negative influence of neurons on the model classification. With the global average pooling (GAP) layer incorporated in the residual shrinkage network structure, we introduce an EEG-based Class Activation Map (ECAM) interpretable method to enable visualization analysis of sample-wise learned patterns to effectively explain the model decision. Extensive experimental results demonstrate that the proposed method achieves the superior classification performance and has found neurophysiologically reliable evidence of classification.

## 1 Introduction

Driver drowsiness is a significant factor leading to traffic accidents as it can cause a serious decline in vigilance, attention, and cognitive ability (Balam and Chinara, 2021). Statistics indicate that fatigue driving may cause up to as much as 20% of all vehicle collisions (Zhang Z. et al., 2022). Effectively predicting and warming about drowsiness in driving can help drivers stay alert before they become drowsy or fall asleep (Perkins et al., 2023). Therefore, developing a reliable and effective drowsiness monitoring system has emerged as a critical priority in preventing traffic accidents and saving lives (Zhang Y. et al., 2022).

Currently, there are a number of reported approaches for detecting driver fatigue or drowsiness, including behavioral (Zhang Z. et al., 2022; Perkins et al., 2023; You et al., 2020), vehicle-based (Perkins et al., 2023; Lan et al., 2024), and physiological (Zhang Z. et al., 2022; Lan et al., 2024) approaches. For example, behavior-based approaches allow to analyze behavioral characteristics of a driver’s face, eyes, or mouth using machine vision technique. These approaches assess alertness level by detecting facial expressions, calculating eye closure time, estimating head posture, and yawning frequency. However, they may be disturbed by lighting conditions and require accurate evaluation of head posture. Vehicle-based approaches focus on detecting drowsiness through vehicle motion and driver handling behavior data (e.g., steering wheel angle, driving acceleration, and vehicle speed) (You et al., 2020). However, they rely on multiple vehicle sensors to monitor driving parameters and may face limitations such as sensitivity and adaptability to environmental factors. In addition, physiological signal-based methods monitor signs of driver drowsiness by analyzing the driver’s physiological signals such as electrocardiogram (ECG), electromyogram (EMG), electrooculogram (EOG), and electroencephalogram (EEG) (Perkins et al., 2023). Drowsiness correlates closely with brain activity, and EEG is the most adaptable and widely used for studying brain functions compared to other physiological signals. Therefore, EEG is often considered the gold standard for detecting driver drowsiness (Lan et al., 2024).

With the rapid development of EEG acquisition devices, an increasing number of researchers have studied EEG-based drowsiness detection (Cui et al., 2022a,c; Wan et al., 2023). Conventional EEG-based drowsiness detection methods laid a solid foundation for ongoing research in this field. To capture the EEG features of interest, they often rely on expert knowledge or priori knowledge (Cui et al., 2022c). For instance, Ogino and Mitsukura (2018) proposed a feature selection method using stepwise linear discriminant analysis and power spectral density features from a single-channel EEG signal. Hu and Min (2018) utilized sample entropy, approximate entropy, spectral entropy, and fuzzy entropy of EEG signals as features for recognizing driving fatigue. In contrast, deep learning (DL) enables end-to-end learning from raw, high-dimensional EEG data without prior feature crafting, achieving remarkable performance (Lawhern et al., 2018; Schirrmeister et al., 2017; Gao et al., 2019). For example, a novel EEG-based spatiotemporal convolutional neural network was developed to detect driver fatigue from multi-channel EEG signals (Gao et al., 2019). Paulo et al. (2021) proposed a deep convolutional neural network for cross-subject calibration-free drowsiness detection based on EEG signals’ spatiotemporal image encoding representations. Di Flumeri et al. (2022) developed a synthetic EEG-based index to detect drowsy events in automotive applications. The proposed MDrow index is proved to be reliable and effective. To further improve classification performance, Li et al. (2023) also attempted to propose an enhanced ensemble deep random vector functional link network for cross-subject fatigue detection performance.

Most of these methods are based on multi-channel EEG, achieving excellent performance. However, multi-channel EEG recording methods are complex to operate, difficult to carry, and have high device costs, all of which hinder their practical application. In addition, many EEG electrodes required to perform an EEG acquisition impose significant restrictions on the user’s mobility on the user’s movement. Compared to multi-channel EEG, only single-channel schedule can offer more practical advantages such as reduced relevant costs, easy signal acquisition, and improved user comfort (Gong et al., 2024). However, due to the individual variability among subjects and non-stationarity in EEG signals and the relatively parsimonious compared to multi-channel EEG (Gong et al., 2024; Liu et al., 2024), designing a zero-calibration drowsiness detection system using only single-channel EEG remains a very challenging task. Additionally, the high sensitivity to artifacts and the low signal-to-noise ratio of EEG signals also exacerbate the difficulty of this task (Cui et al., 2022c).

To address these limitations, some researchers recently focus on single-channel EEG-based drowsiness detection using DL methods (Liu et al., 2024; Fahimi et al., 2019; Ding et al., 2019; Reddy et al., 2024). For example, Liu et al. (2024) explored a single-channel EEG-based self-training semi-supervised method to transform the unlabeled data into pseudo-labeled data and combine the fuzzy entropy feature for fatigue driving detection. Fahimi et al. (2019) proposed an end-to-end deep convolutional neural network (CNN) to detect attentive mental states using a single-channel EEG. To design a portable wearable EEG device for recognizing driver drowsiness, Ding et al. (2019) designed a DL model with a cascaded CNN and an attention mechanism. Reddy et al. (2024) proposed an effective hybrid DL model for single-channel EEG-based subject-independent drowsiness detection, which combined discrete wavelet long short-term memory and convolutional neural networks. To extract task-relevant discriminative features, Divvala and Mishra (2024) proposed a DL-based attention mechanism to recognize drowsiness state. However, these DL models in previous works are often regarded as “black-box” classifiers due to their lack of interpretability while maintaining accuracy (Gao et al., 2023b; Cui et al., 2021). Hence, it is crucial to develop an inherently interpretable DL model to address this limitation.

Some efforts had been made to explore interpretable models to understand the decision-making process based on the learned characteristics of input EEG. For instance, Cui et al. (2021) proposed a CNN with long short-term memory (LSTM) to visualize the common EEG features learned from single-channel EEG signals for driver drowsiness classification. Furthermore, Cui et al. (2022b) proposed an interpretable DL model with compact CNN structure to explain what features the model had learned from single-channel EEG signals. However, there is usually a trade-off between interpretability and performance (Wang et al., 2020). Both inherently interpretable methods were designed at the cost of performance degradation as they did not adequately mine and extract the salient features implicit in raw single-channel EEG signals.

To address the above issues, we propose a novel interpretable residual shrinkage network (ID3RSNet) for driver drowsiness detection from single-channel EEG signals. The framework consists of a base feature extractor (BaseFE), residual shrinkage building unit (RSBU) with soft thresholding (ST), a global average pooling (GAP) layer, and a fully connected layer with weight freezing (FC-WF). First, the base feature extractor (BaseFE) extracts the essential features of EEG frequencies. Second, a residual shrinkage building unit (RSBU) with channel-wise thresholds is adopted to improve the feature learning ability. Automatic feature extraction is achieved by applying soft threshold denoising and attention mechanism within the residual shrinkage neural network. Then, a following GAP layer is added to avoid overfitting and improve generalization ability. In addition, a regularization method of weight freezing is applied in the FC layer to effectively suppress the negative influence of some input neurons on the model classification. Based on the designed residual shrinkage network structure, we can use an EEG-based class activation map (ECAM) interpretation method to visualize neurophysiologically common patterns learned from single-channel EEG signals for classification decision.

The main contributions in this work are as follows:

1. To the best of our knowledge, we propose the first end-to-end inherently interpretable deep residual shrinkage network framework to achieve automatic feature extraction and enhance the feature learning ability for driver drowsiness detection. With only single-channel EEG used, the framework has greater potential practical value.
2. With the inherently interpretable model framework designed, we propose a class activation map interpretation method for raw single-channel EEG signals to reveal neurophysiologically common patterns in terms of the driver’s mental state.
3. Extensive experiments with leave-one-subject-out cross-validation (LOSO-CV) demonstrate the effectiveness of the proposed method with reliable classification evidence discovered. This work also provides insight into the development of portable single-channel EEG devices with interpretable neural network for driver drowsiness detection.

The study is organized as follows. Section 2 is the materials and methods. Section 3 is the experimental results. Section 4 is the discussion and future works. The last section is conclusion.

## 2 Methods

### 2.1 Overview

**Figure 1:**
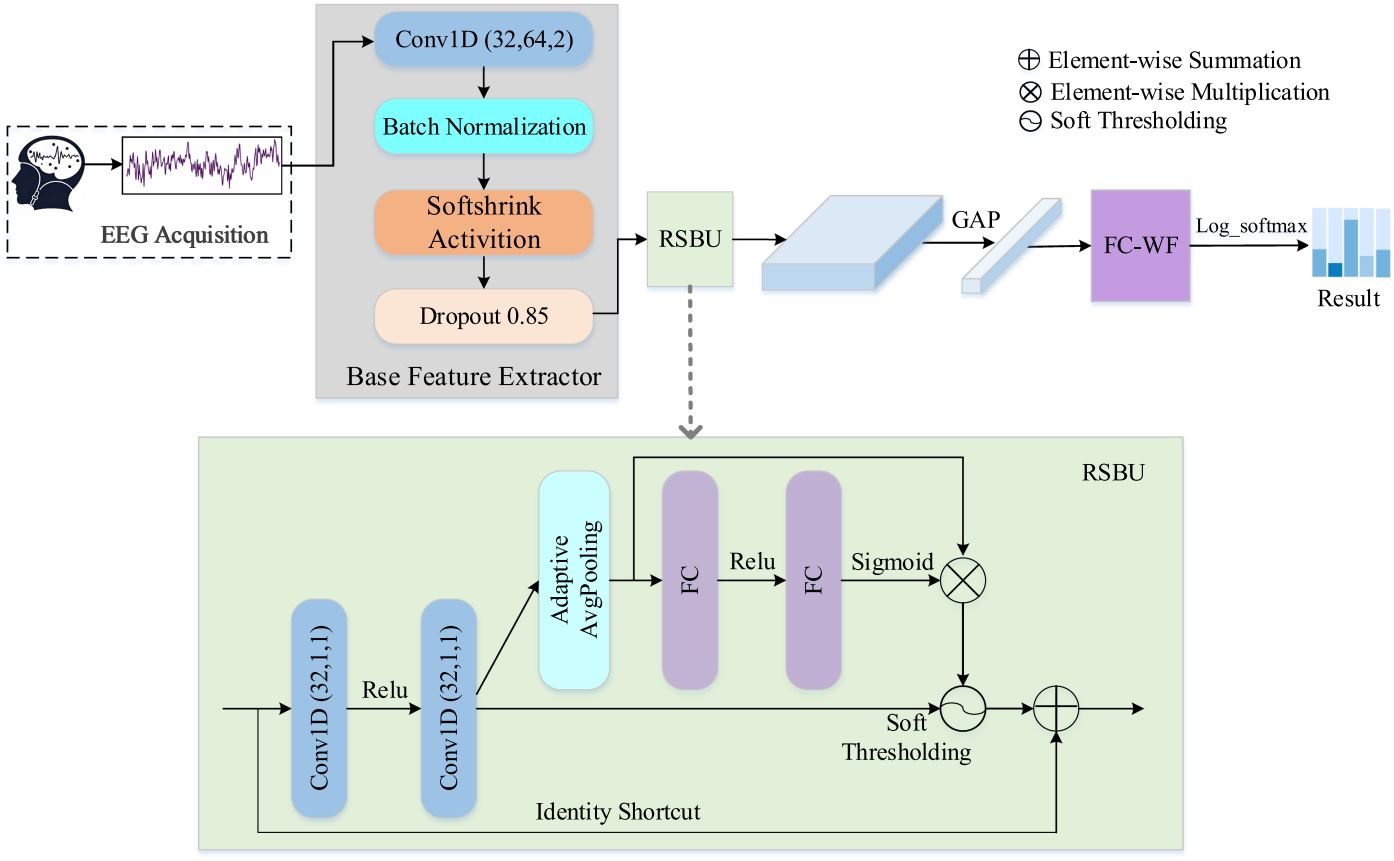
Overall architecture of the proposed interpretable network framework.

To accurately characterize drowsiness-related patterns and enhance feature representation from non-stationarity EEG signals with high randomness and low signal-to-noise ratio, we propose a novel interpretable residual shrinkage network (ID3RSNet) framework shown in Figure 1, whose framework mainly consists of three modules: base feature extractor (BaseFE), residual shrinkage building unit (RSBU), and classification. First, the BaseFE extracts the essential features from a 3-s EEG signal. Following BaseFE, we propose a residual shrinkage building unit with channel-wise thresholds (RSBU-CW) to enhance the quality of the extracted features and achieve automatic extraction of important features, which helps to improve classification performance. Then, a global average pooling layer is added as a key component in the inherently interpretable model structure, which helps to avoid overfitting and improve the generalization ability. In addition, a regularization method of weight freezing in the FC layer is applied to effectively suppress the negative influence of some input neurons on the model classification. To provide trustworthy interpretation classification for the proposed ID3RSNet, we introduce an EEG-based class activation map (ECAM) interpretation method to reveal neurophysiologically task-related patterns.

### 2.2 Network of the proposed model

#### 2.2.1 Base feature extractor

Considering that convolutional neural networks (CNNs) are widely utilized to capture the EEG features of time series data (Hu et al., 2023), we designed a base feature extractor (BaseFE) to extract the key EEG features. The BaseFE network is designed in a shallow network structure. As shown in Figure 1, the S-CNN module contains five layers: a 1-D convolution layer, a batch normalization layer, a Softshrink activation layer, and a dropout layer.

The Conv1D (32, 64, 2) in Figure 1 refers to 1-D convolution layer with 32 filters, a kernel size of 64, and a stride of 2. Setting the kernel size to 64, half of the 128 Hz data sampling rate, enables the model to extract the EEG frequency features in the range above 2 Hz. Then, a batch normalization layer and a Softshrink activation layer are followed. The batch normalization is utilized to normalize small batches across each feature dimension, effectively mitigating internal covariate shifts (Ioffe and Szegedy, 2015). Softshrink function is a non-linear activation function that is mainly used for sparse representation of EEG data and noise suppression. To mitigate model overfitting, a dropout layer is introduced in the following layer.

#### 2.2.2 Residual shrinkage building unit

Inspired that deep residual shrinkage networks achieved high fault diagnosing performance in vibration signals (Zhao et al., 2020), we introduce the residual shrinkage network to improve the feature learning ability from single-channel EEG signals. Both the attention mechanism and automatic soft thresholding are integrated into the residual network to adaptively eliminate redundant information and selected the most discriminative useful features during feature learning. This residual structure is to prevent vanishing gradients and exploding gradients in deep network.

Based on the fact that the importance between each channel of the features learned from the EEG signal is different, we use a residual shrinkage building unit with channel-wise thresholds (RSBU-CW) in this work. In particular, the squeeze and excitation (SE) network is adopted to obtain a set of thresholds related to the individual channels by modeling the inter-dependencies between the features (Hu et al., 2020). Soft thresholding incorporated in the RSBU structure can adaptively eliminate redundant information and select highly discriminative features.

As is shown in Figure 1, two convolutions Conv1D (32, 1, 1) with a kernel size of 1 and a stride of 1 are implemented in this block. Assuming that the BaseFE module generates a feature map *I* ∈ ℝ*^L × T^*, we apply two convolutions operations (*Conv*1 and *Conv*2) to *I* to obtain *U* (*U*∈ *ℝ^N × T^*) in Equation 1:


(1) $$U=\mathrm{Conv}2\left(\mathrm{Conv}1\left(I\right)\right)$$

where *T* represents the length of *U*, and *N* represents the total number of features.

Then, the feature map *X* is squeezed to a 1-D vector using an absolute operation and an adaptive average pooling layer. The excitation operation is used to capture the correlation between the individual channels, which can retain the channels with the most useful feature information and suppress the channels with less feature information (Eldele et al., 2021). In this study, two fully connected (FC) layers are added for information aggregation. The first FC layer, followed by ReLU, aims to reduce dimensionality, while the subsequent layer, followed by a sigmoid function, aims to raise dimensionality. The output of the FC layers is scaled to between (0, 1) with the scaling parameters. And the scaling parameter is described in Equation 2:


(2) $$\sigma_{r}=\frac{1}{1+e^{-z_{r}}}$$

where *z_r_* represents the feature at the *r*th neuron, and *σ_r_* represents the *r*th scaling parameter. To make the soft threshold positive and not too large, the scaling parameter *σ_r_* is multiplied with the average absolute value of *U_r_* to obtain the threshold. The threshold used in RSBU-CW is expressed in Equation 3:


(3) $$\tau_{r}=\sigma_{r}\cdot \underset{i}{\mathrm{average}}\;|U_{i,r}|$$

where *τ_r_* represents the threshold for the *r*th channel of the feature map, and *i, r* represent the indexes of length and channel of the feature map *U*, respectively. Rather than the artificial design of filters by experts, the threshold is automatically determined through the SE attention mechanism. The mechanism is that the CNN automatically conducts filter learning and transforms the original data to a new space for soft thresholding. The soft thresholding function formula can be expressed in Equation 4:


(4) $$y=\{\begin{array}{cc} x-\tau , & x>\tau \\ 0, & -\tau \leq x\leq \tau \\ x+\tau , & x<-\tau \end{array}$$

In this study, *τ* is a parameter that can be learned by means of automatic learning. The thresholds can be kept in a reasonable range, thereby preventing the output of soft thresholding being all zeros. Finally, the input *I* is combined with the enhanced features O learned from the residual unit by adding an identity shortcut connection. The final output of this residual unit is expressed in Equation 5:


(5) P=I+O

#### 2.2.3 GAP and FC-WF

To avoid overfitting, the GAP is used to replace the FC layer, and the number of model parameters is further reduced significantly. The GAP reduces the *N* dimensional filtered signal to *N* feature points through an average pooling operation. It not only helps to improve generalization capability of the network but also allows an EEG-based Class Activation Map (ECAM) interpretable method to reveal learned patterns in terms of the driver’s mental state.

In the fully connected layer, the weight freezing method is introduced to suppress the update of some learnable parameters by freezing some weights during the backpropagation process. Assuming the features inputted into the FC layer are denoted as **F =** {**f**_1_,…, **f***_n_*}, where **F**∈ℝ*^L × N^*. *L* denotes the size of the mini-batch, and *N* is the feature dimension. **W***_n_* denotes the weights and the vector of inputs of the FC layer, respectively. Weight freezing is proposed as a regularization method to improve classification accuracy, which can be implemented in Equation 6:


(6) $$W_{n}=W_{n}-K⊙\left(\eta \cdot \left({\tilde{z}}_{m}-z_{m}\right)f_{n}\right)$$

where **K** is a mask matrix with identical dimensions to **W***_n_*, and the elements of **K** are uniformly distributed within the range [0,1]. ⊙ is the element-wise multiplication and *η* denotes the learning rate of the optimizer.

When an element is masked, it cannot be updated during backpropagation. Here, we set the threshold value t (=0.2) of **K**, which determines the number of frozen parameters in **W***_n_*. In the FC layer, we utilize the weight freezing method not only to make sparse connections but also to improve classification accuracy. Finally, the cross-entropy loss function with label smoothing regularization of parameter *α* (=0.1) is utilized to optimize the classification model, which can be described in Equation 7:


(7) Loss=−∑c=1Cyc1−α+αC]log(pc

where *y_c_* is the true label, *p_c_* is the predicted probability of the model for the class *c*, and *C* is the number of classes.

### 2.3 ECAM interpretation method

Class activation map (CAM) is a heatmap that contains classification information, and it can highlight regions most pertinent to a specific class through region-level feature highlighting (Zhou et al., 2016). The designed residual shrinkage network structure is combined with an EEG-based class activation map (ECAM) interpretation technique, which allows to visualize neurophysiologically common patterns learned from single-channel EEG signals for classification decision.

**Figure 2:**
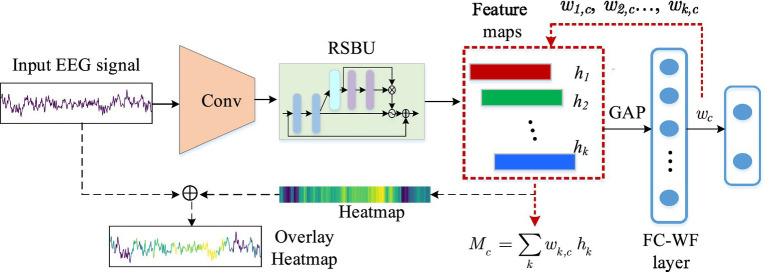
Process of the interpretation method over the input EEG signal.

The process of the interpretation method over the input signal is shown in Figure 2. The SE residual block is utilized to pay more attention to the significant channel information in the feature map by adaptively adjusting the weights of each channel. Each channel correlates with a different feature and contributes differently to each output classes. In this study, the class activation weights (CAWs) are the weights of the FC layers, leading to different weights for each feature map channel. The generated CAM will give more prominence to features that contribute significantly to the model’s decision-making, while less relevant features will be well suppressed. The CAWs of each channel allow us to visualize discriminative regions of the EEG signals, which are considered as the basis for classification.

Assuming the 3-s EEG input signal is *X* = {*x_i_*} (*i* = 1,2,…,384), and the activation of the *k*th node in the output layer of the residual shrinkage unit is *h_k,j_*. Where *k* = (1,…,*N*) with *N* = 32 representing the number of features, and *j* = (1,2,3,…,*T*) with *T* representing the length in time dimension. Since the first convolution layer reduces the raw signal of length 384 to 192, the final output of the residual shrinkage unit is a feature map of 192 (*T*)*32 (*N*). Let $m_{k}^{g}$ denotes the feature activation of the *k*th node output by the GAP layer, and it can be computed as follows:


(8) $$m_{k}^{g}=\frac{1}{T}\underset{j}{\sum }h_{k,j}$$

Suppose $m_{c}^{d}$ denotes the activation of the node corresponding to class *c* in dense layer. Here, *c* = 0 or 1 denotes the state of alert or drowsy, respectively. We compute $m_{c}^{d}$ as follows:


(9) $$m_{c}^{d}=\underset{k}{\sum }w_{k,c}m_{k}^{g}+b_{c}$$

where *ω_k,c_* represents the FC-WF layer’s weight associated with class *c* for the node activation $m_{k}^{g}$ , and *b_c_* is the bias of class c in the FC-WF layer. It is also the CAW utilized in this interpretation method. The $m_{c}^{d}$ is considered as the final activation of the network. *M_c_*(*j*) denotes the activation map for class *c*. From Equations 8, 9, *M_c_*(*j*) is computed in Equation 10:


(10) $$M_{c}\left(j\right)=\underset{k}{\sum }w_{k,c}h_{k,j}$$

where *b_c_* and 1∕*n* are neglected for simplicity. Then, the heatmap is further normalized by z-score. We can have the heatmap *M_c_*, where *M_c_* = (*z_1,c_*, *z_2,c_*,…, *z_T,c_*).

Similar to the CAM method (Zhou et al., 2016), the heatmap indicating the classification decision of the model is obtained by upsampling *M_c_*(*j*) to the same length as the input signal. The upsampling is to use an equal interpolation method to fill the elements based on the original activation length of 192. According to the network structure, the convolution and pooling layers of BaseFE module reduce the temporal dimension to 1/2 of the input signal, and the residual shrinkage block does not change its temporal dimension. Therefore, the heatmap can be restored to the original length of 384, which is same as the input signal, by duplicating and filling each element *z_j,c_* two times. The final heatmap is obtained in Equation 11:


(11) $$M_{c}=\left(\underset{2}{\underset{︸}{z_{1,c},\ldots ,z_{1,c,}}}\ldots ,\underset{2}{\underset{︸}{z_{n,c},\ldots ,z_{n,c}}}\right)$$

### 2.4 Methods for comparison

In this section, there are several leading single-channel EEG-based baseline methods for comparison, including the conventional machine learning methods and state-of-the-art deep learning methods.

1. Conventional methods: The power feature of EEG bands is one of the crucial features for EEG drowsy state recognition (Cui et al., 2021). We calculate the relative power (delta, theta, alpha, and delta bands) from the Oz channel signal using the Welch’s method. Different conventional classifiers are tested, which include k-nearest neighbors (KNN), Random Forest (RF), Gaussian Naive Bayes (GNB), and SVM.
2. EEGNet: EEGNet was designed as a compact CNN model (Lawhern et al., 2018). We opt for EEGNet-8,2 over EEGNet-4,2 due to its higher classification accuracy. Despite its compact network, EEGNet-8,2 can achieve the state-of-the-art performance in various EEG recognition tasks.
3. ShallowConvNet (Schirrmeister et al., 2017): ShallowConvNet is a shallow CNN consisting of temporal convolution, spatial convolution, and pooling layers.
4. DeepConvNet: In addition to the Shallow CNN model, Schirrmeister et al. (2017) proposed another effective deep CNN model (DeepConvNet) to capture discriminative EEG features for motor imagery classification.
5. CNN-LSTM: CNN-LSTM was designed to recognize subject-independent drowsiness from single-channel EEG and provide interpretable analyze for classification (Cui et al., 2021).
6. CompactCNN: CompactCNN is proposed as an interpretable DL model, which applies the CAM method to visualize EEG common features learned from single-channel EEG (Cui et al., 2022b).
7. TSANet: TSANet is a deep neural network model based on temporal-spectral fused and attention which is originally used for automatic sleep staging from single-channel EEG (Fu et al., 2023).

## 3 Experimental results

In this section, we first describe the widely used dataset, our experiment, and evaluation metrics. Then, we conduct extensive experiments and present the performance of our model in comparison with the strong baselines.

### 3.1 Data description

In this study, we use a public sustained-attention driving task (SADT) dataset to explore driver drowsiness detection (Cao et al., 2019). The EEG data were collected from 27 participants (ranging in age from 22 to 28) with headset EEG of 32 electrodes at 500 Hz. The driver drowsy state was induced through a 90-min nighttime driving simulation which is conducted in a VR-based driving simulator. During this procedure, lane departure events occurred when the car was drifted from the center lane either to the left or right. Participants were asked to promptly steer the car back to the center lane as soon as the events occurred. The drowsy degree was quantitatively assessed on the basis of the subjects’ reaction times to these departure events. By analyzing subjects’ reaction time to these events, it was able to gauge their level of drowsiness.

The recorded signals were first filtered by 1–50 Hz band-pass filters and then processed by artifact rejection. Cui et al. (2022b) further preprocessed the EEG signals by down-sampling to 128 Hz. Then, they selected and labeled the samples to generate a preprocessed version of the dataset. The samples were extracted for each EEG trail at 3-s length before the car deviation event (Gong et al., 2024). Notably, studies have shown that drowsiness is associated with EEG power spectrum in the theta and/or alpha band (Joutsiniemi et al., 1995). After attempting to find good choices of the EEG channel and power spectrum features for assessing the drowsiness-related EEG dynamics, it was found that the Oz channel is the most effective channel and its power spectrum features in the theta and alpha band have good distinguishing ability (Pal et al., 2008). Therefore, we select the Oz channel data to find the most discriminative features for identifying drowsiness from alert samples from the single-channel EEG signals in the study. The dimension of each sample is 1 (Oz channel) × 384 (sample points).

To ensure sufficient training and testing samples, each subject containing at least 50 samples for each state was selected for the dataset. Finally, 2,952 samples from 11 different subjects were collected to produce an unbalanced dataset (Cui, 2021a) for the real situation, which is described in Table 1. In addition, they also generated a balanced dataset including 2022 samples which has been uploaded online (Cui, 2021b). In this study, we view the balanced dataset as an ideal training dataset for the models and utilize the unbalanced data of each subject to evaluate the training model.

**Table 1:** Number of samples in the unbalanced dataset.

Subject ID	Sample number	
	Alert	Drowsiness
1	94	96
2	363	66
3	75	180
4	118	74
5	161	112
6	83	116
7	51	103
8	238	132
9	243	157
10	192	54
11	113	131
Total	1,731	1,221

### 3.2 Experimental settings and evaluation metrics

In our experiment, we conduct comparisons on a desktop computer with an Intel(R) Core(TM) i5-12600KF CPU and a NVIDIA GeForce GTX 1080 Ti graphics card. All the codes were implemented in Python 3.6, and our model along with the baseline methods was implemented using the PyTorch Library. For EEGNet, ShallowConvNet, and DeepConvNet, we made a slight modification to their original models. Since each second convolutional layer in these three models is designed to extract features from multi-channel EEG, here we made a slight modification in each second convolutional layer with a 1 × 1 kernel used to adapt for single-channel EEG signals, which can enhance spatial feature extraction and the feature representation ability. We utilized the Adam optimization method with a learning rate of 0.001 and set the batch size as 50.

For cross-subject driver drowsiness detection, the leave-one-subject-out cross-validation (LOSO-CV) is conducted to evaluate our model’s effectiveness on a widely used dataset of 11 subjects (Autthasan et al., 2022). In each fold of LOSO-CV, the EEG data samples from one subject are set up as a testing set for testing, and the data samples from all the other subjects are set up as a training set for training. This iterative process is repeated until each subject has been tested once as the test subject.

We use accuracy as an evaluation metric of our method in our experiment. As the metric of the F1-score considers both the precision and recall of the classification model, it is generally considered to be the most suitable metric for the unbalanced dataset. Therefore, we also adopt the F1-score as an evaluation metric on the unbalanced dataset. The metrics of sensitivity test and deletion test are also used to evaluate the interpretation of interpretable models.

### 3.3 Results and comparison

#### 3.3.1 Mean accuracy comparison on the balanced dataset

In this section, we compared the classification accuracy of five deep learning models tested on the balanced dataset for a standard evaluation. Each model was trained from 1 to 20 epochs, with network parameters randomized for each iteration. We conducted each evaluation by repeating each model on every subject 10 times, resulting in 110-fold for each epoch (10 times × 11 subjects).

**Figure 3:**
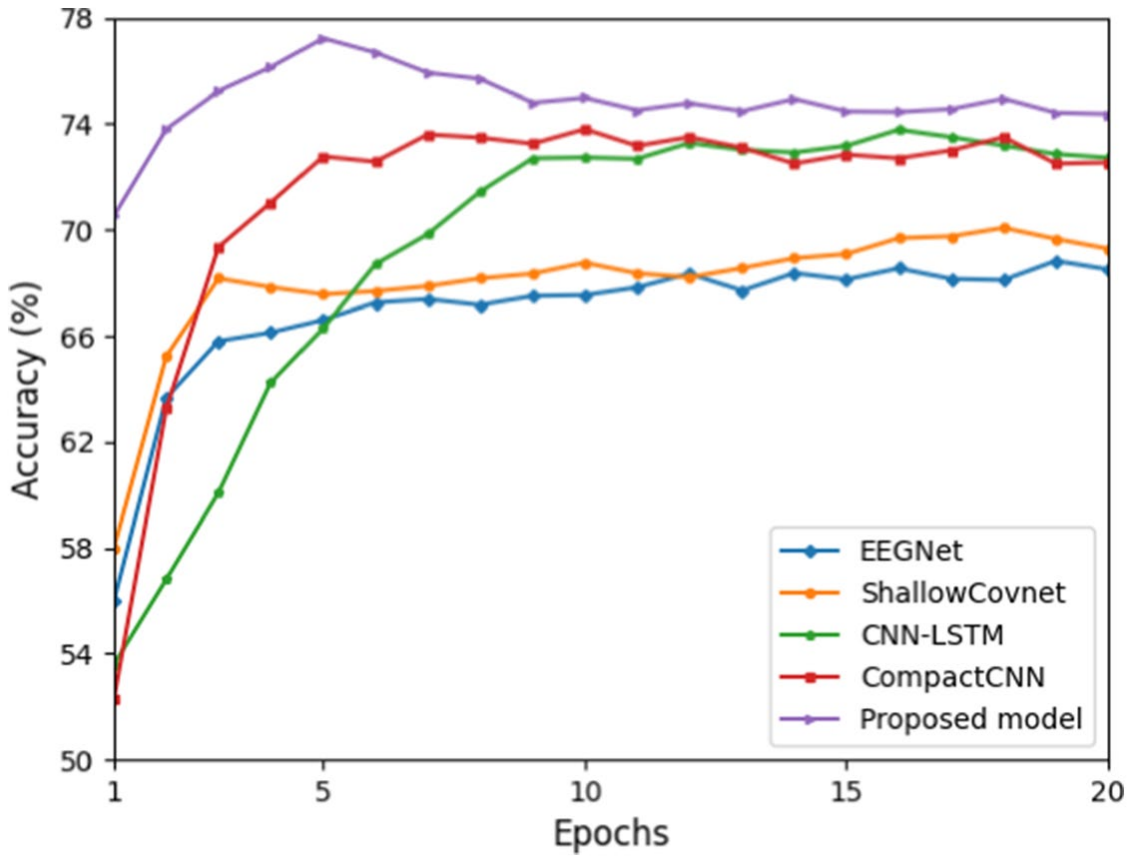
Average cross-subject classification accuracies of the proposed model and four benchmark deep learning models for training epochs from 1 to 20.

Figure 3 shows that the proposed ID3RANet outperforms other benchmark DL models. After 5 epochs of training, it achieves a peak mean accuracy of 77.16% with the fastest convergence speed. In the rest of the first 20 epochs, its mean accuracy stabilizes at above 74.42%, outperforming the other four models. In contrast, the CNN-LSTM model exhibits slower convergence, reaching an average accuracy of 73.78% after 16 epochs of training. The CompactCNN and CNN-LSTM models achieve the overall higher mean accuracies than both models of the EEGNet and ShallowCovnet, and their highest mean accuracies in the first 20 training epochs are 73.80 and 73.78%, respectively. Even though the CompactCNN model reaches a good performance rapidly in the first 10 epochs and stabilizes at approximately 72.20%, it is lower than that of the proposed model. The results indicate that the proposed method can better capture the class-discriminative EEG features for drowsiness detection from single-channel EEG signals.

#### 3.3.2 Comparison results on the unbalanced dataset

In this section, we show the accuracies and F1 score comparison results of different methods tested on the unbalanced dataset, which is closer to the real-life scenarios. As shown in Tables 2, 3, comparing to all baseline models (including conventional methods and six deep learning methods), our proposed method achieves the better performance, due to its enhanced features learning ability. Moreover, we can also draw the following conclusions. First, it can be found that conventional methods (KNN, RF, GNB, and SVM) generally achieve relatively inferior classification performance compared to deep learning-based approaches except EEGNet. This demonstrates that the end-to-end deep learning methods can learn more task-relevant discriminative features for classification. Second, our method improves the average classification accuracy of approximately 4.85% on average and 7.8% on maximum in comparison with the deep learning methods such as CompactCNN and TSANet. This demonstrates that our method can achieve effective mining of important information implicit within single-channel EEG signals by effectively exploiting attention and soft thresholding in the residual shrinkage network. Furthermore, our method achieves an average F1-score almost 1.38% higher than the best TSANet method, all of which demonstrates its effectiveness and potential practical value. Finally, multiple optimal or suboptimal classification results (optimal results bolded in black) are achieved in individual test for each subject, which also proves its strong generalization ability for classifying cross-subject drowsy states from single-channel EEG signals.

**Table 2:** Comparison of different methods for cross-subject classification accuracy on the unbalanced dataset (%).

Methods	Subject ID											Avg. Acc.
	1	2	3	4	5	6	7	8	9	10	11	
KNN	71.05	41.96	36.86	67.71	69.60	67.34	68.83	62.16	76.00	83.33	61.89	64.25
RF	70.53	40.33	35.29	68.75	72.53	73.87	71.43	63.24	77.25	86.59	56.15	65.09
GNB	77.89	48.48	32.16	73.96	76.92	72.36	70.78	45.95	76.25	85.77	60.66	65.56
SVM	76.32	44.76	34.12	68.75	74.36	71.86	74.68	57.03	82.50	90.24	63.11	67.07
EEGNet	80.00	46.15	32.94	71.88	75.09	73.37	71.43	52.16	83.75	85.77	63.52	66.92
ShallowConvNet	**85.26**	45.92	32.94	59.90	67.40	80.40	**78.57**	42.97	84.00	89.02	73.36	67.25
DeepConvNet	72.63	**59.21**	30.59	59.90	61.90	75.38	75.97	70.54	81.00	**92.68**	57.38	67.02
CNN-LSTM	77.89	42.89	**52.55**	66.67	78.02	79.40	75.97	72.43	81.25	86.99	75.41	71.77
CompactCNN	78.42	53.85	52.16	64.58	78.39	77.39	72.73	**72.97**	89.25	84.15	72.95	72.44
TSANet	84.21	48.25	46.67	71.88	78.75	**86.43**	77.92	63.24	**91.25**	90.65	72.54	73.80
Ours	83.68	50.82	49.41	**76.56**	**86.81**	79.40	77.27	69.73	89.50	82.11	**76.64**	**74.72**

**Table 3:** Comparison of different methods for cross-subject classification F1-scores on the unbalanced dataset (%).

Methods	Subject ID											Avg. F1.
	1	2	3	4	5	6	7	8	9	10	11	
KNN	72.08	23.85	29.69	65.17	50.89	67.01	78.18	62.96	74.74	62.39	57.14	58.55
RF	70.83	24.26	26.01	65.91	56.14	73.74	79.82	64.95	75.86	69.72	51.14	59.85
GNB	76.67	**28.48**	11.28	73.12	64.41	71.20	79.82	56.90	76.31	66.67	45.45	59.12
SVM	75.41	26.17	17.65	64.71	56.25	69.89	82.19	62.23	80.45	76.92	51.61	60.32
EEGNet	81.77	24.92	15.08	65.90	56.05	72.13	82.14	57.59	81.20	74.07	43.02	59.44
ShallowConvNet	82.41	25.42	53.38	53.13	37.58	73.96	82.57	56.51	77.18	74.02	50.28	60.58
DeepConvNet	65.79	10.26	3.29	38.40	13.33	73.80	81.59	56.57	70.08	**81.25**	37.35	48.34
CNN-LSTM	76.40	25.53	**64.52**	65.22	75.61	80.75	82.13	64.58	79.34	72.88	**78.57**	69.59
CompactCNN	76.57	26.67	60.89	65.66	74.46	78.05	78.35	**70.23**	87.09	67.77	73.60	69.03
TSANet	**83.87**	23.97	51.77	70.97	65.88	**87.78**	**83.65**	65.66	**89.55**	78.10	67.63	69.89
Ours	83.06	26.48	58.25	**73.37**	**84.07**	79.40	82.59	66.27	87.35	65.63	77.47	**71.27**

#### 3.3.3 Ablation experiments of the proposed modules

To verify the effectiveness of each module of our model, we conduct ablation experiments tested on the unbalanced dataset. Note that our proposed is made up of base feature extractor (BaseFE), residual shrinkage building unit (RSBU) with soft thresholding (ST), GAP, and a fully connected layer (FC) with weight freezing (WF). Specifically, we derive six model variants as follows.

1. *BaseFE*: The base feature extractor module only.
2. *w/o RSBU*: The model removes the residual shrinkage building unit with soft thresholding.
3. *w/o ST*: The model removes the soft thresholding.
4. *w/o GAP*: The model removes the global average pooling layer.
5. *w/o WF*: The model removes the weight freezing from the fully connected layer.
6. *ID3RSNet*: The model includes each module of proposed model in this study.

**Figure 4:**
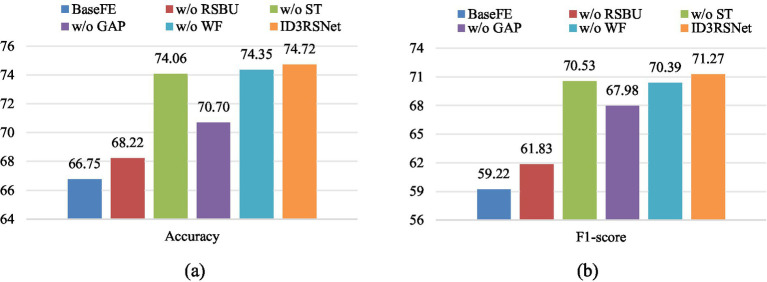
Result of ablation experiments on unbalanced data settings. The ‘w/o’ represents ‘without’. The values of accuracy and F1-score are percentile. **(A)** Accuracy, **(B)** F1-score.

From the results of ablation study shown in Figures 4A,B, we can probably summarize as follows. First, the model variable *w/o RSBU*, which completely removes the residual shrinkage building unit module, yields almost the worst results. This suggests that the channel-wise residual shrinkage building unit contributes significantly to enhance the features learning ability and model classification performance. Furthermore, in comparison with the model variable *w/o ST* and *w/o RSBU*, we know that the residual SE block contributes more to boosting feature representation. Second, the model variable *w/o GAP* achieves obviously inferior results, indicating that the GAP can effectively reduce overfitting and improve generalization capability of the proposed model. Finally, the proposed *ID3RSNet* achieves superior classification performance with each module integrated, which shows the necessity and effectiveness of each module.

#### 3.3.4 Evaluation of sensitivity test and deletion test

**Figure 5:**
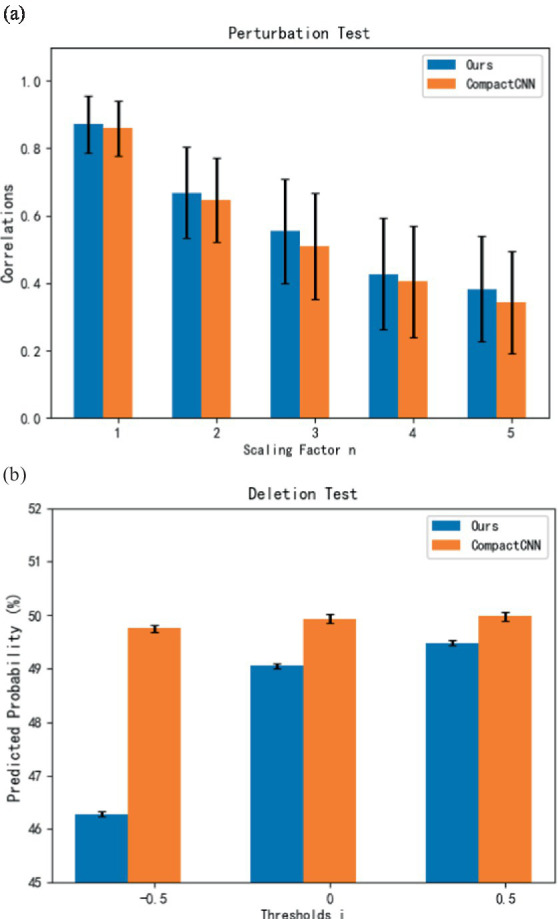
Evaluation results of sensitivity test and deletion test for interpretable models. **(A)** Sensitivity test. **(B)** Deletion test.

For sensitivity tests, we randomly selected 50 samples of each class from each subject in the unbalanced dataset and thus have in total 11 (subjects) × 2 (classes) × 50(samples) = 1,100 samples for evaluation (Cui et al., 2023). Inspired by the sensitivity test proposed by Cui et al. (2023), we adopted the non-linear perturbation (sine wave combined with noise) and adjusted the perturbation strength dynamically with a ranged perturbation scale of the input sample. It is assumed that the perturbations will not cause the sample to deviate significantly from its original distributed. The sensitivity test is performed on the original contribution heatmap to reflect the best correlation obtained between the perturbed batches and the model output. Here, we set the scaling factor *n* of perturbation to 1–5 and calculate the Pearson correlation coefficient (PCC) between the original heatmap and perturbed heatmap as a quality metric of the contribution map. From Figure 5A, it can be observed that our method achieves higher average correlation coefficients in the sensitivity tests compared to the CompactCNN baseline (Cui et al., 2022b). It indicates that the contribution maps generated by our model have higher stability and consistency under different input perturbations.

According to the deletion test proposed in the reference (Petsiuk et al., 2018), we also use the metric of deletion test in this study. In this test, we ranked the sampling points of the input sample according to their descending order based on their values in the contribution map (Cui et al., 2023). By setting the sample threshold for deleting values in the heatmap, we calculated the predicted probabilities when the corresponding points were removed from the sample by setting their values to zeros. The indicative of a high-quality contribution map is a sharp drop of the predicted probabilities for the corresponding class. From Figure 5B, it can be seen that our method achieves lower average values of predicted probabilities with the different sample thresholds compared to the CompactCNN baseline. It indicates that the interpretation of our method is more effective than the CompactCNN due to the removal of important features.

#### 3.3.5 Interpretation on the learned patterns from single-channel EEG signals

**Figure 6:**
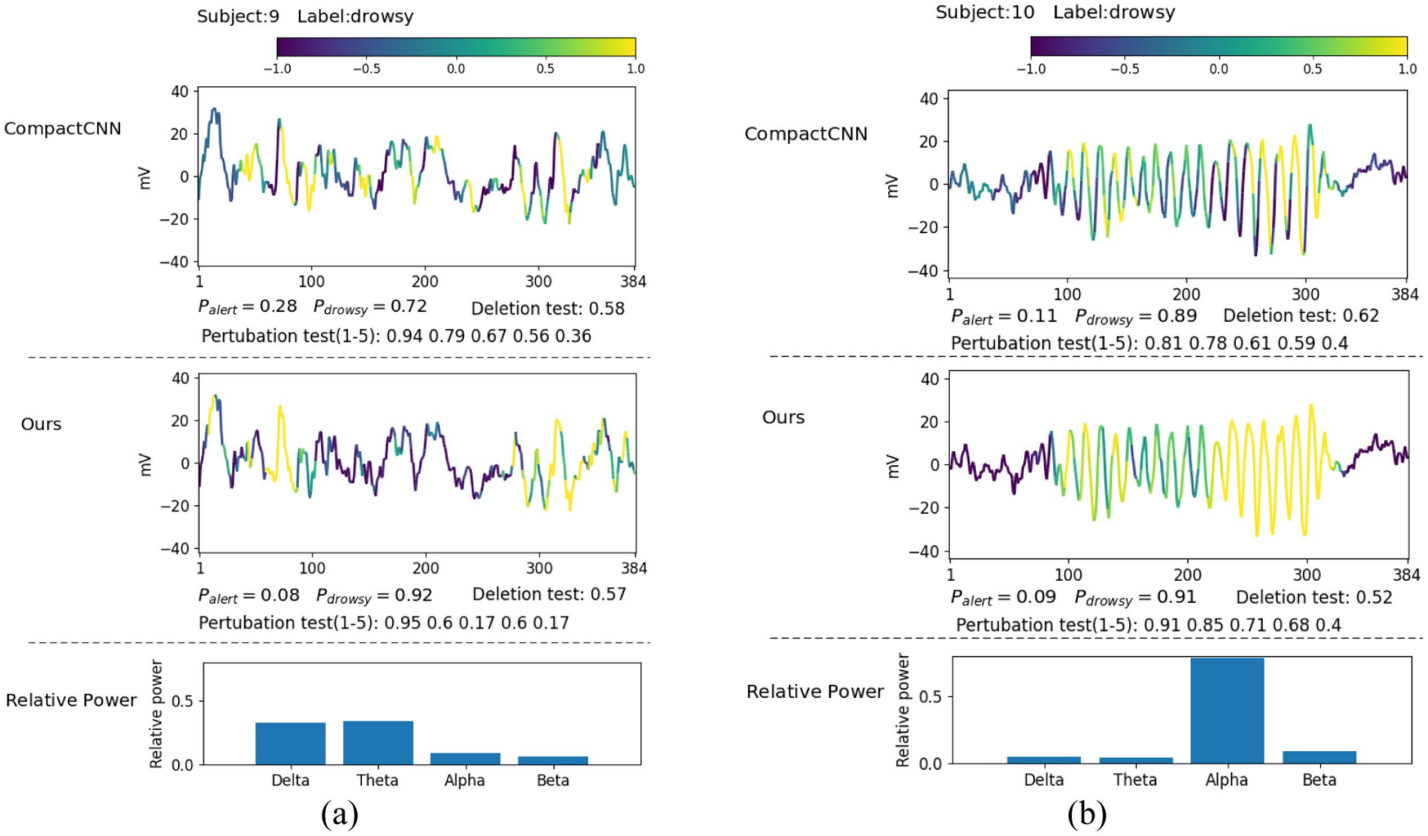
Illustrative comparison of the visualization of learned patterns on correctly classified drowsy samples: **(A)** theta-delta burst; **(B)** alpha spindle.

**Figure 7:**
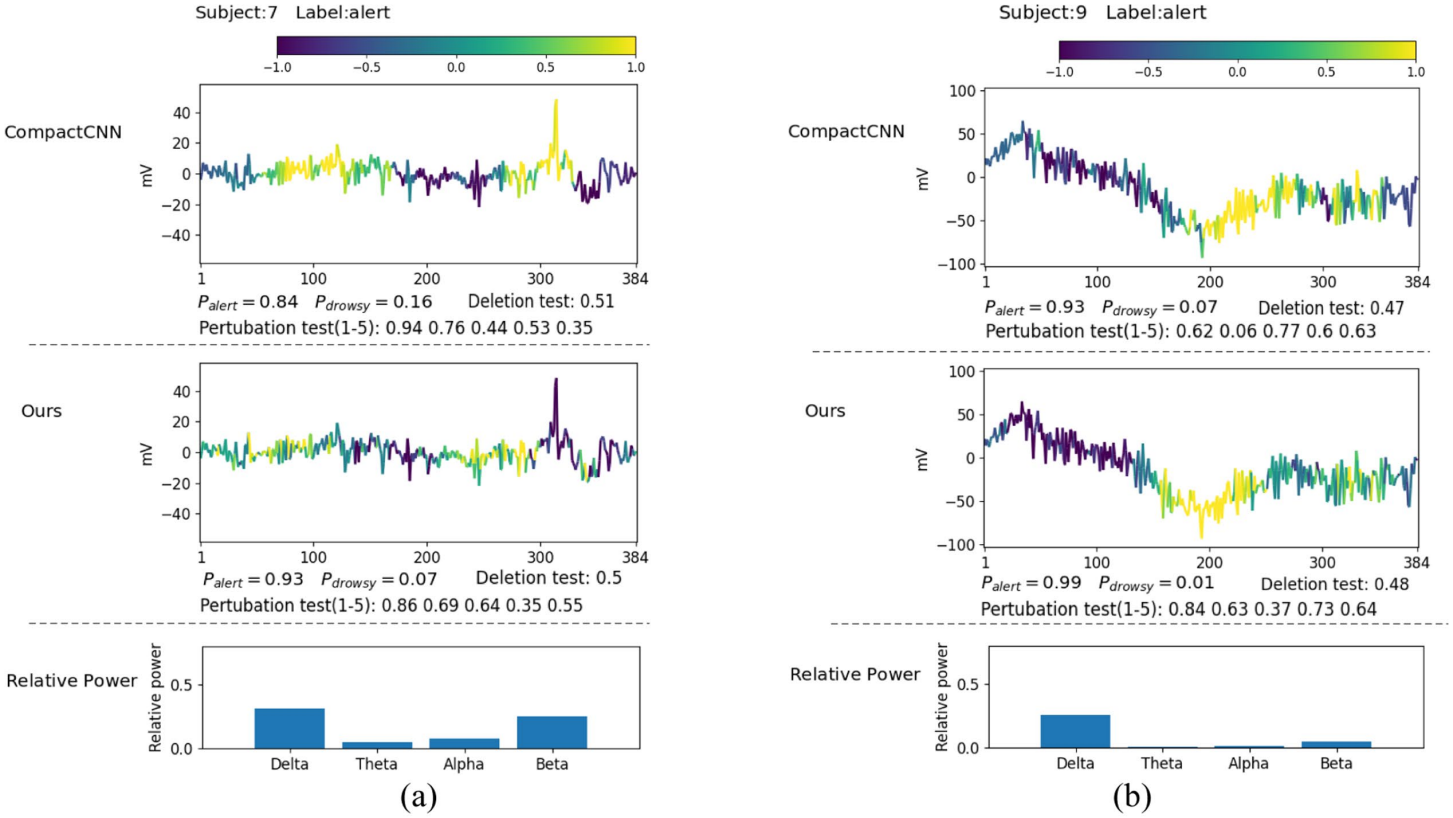
Illustrative comparison of the visualization of learned patterns on correctly classified alert samples: **(A)** beta rhythm; **(B)** delta rhythm.

In this section, we explore what EEG patterns our proposed ID3RSNet has learned using the proposed ECAM interpretation method, which is described in Section 2.3. From the generated heatmap of EEG amplitude fluctuation and the bar graph of relative power, we can explain the most discriminative features learned as evidence of the model classification. To verify the reliable interpretation of the model decision, we compare our method with the leading interpretable deep learning method CompactCNN (Cui et al., 2022b). Figures 6, 7 show the visualization of some representative EEG samples labeled as drowsy and alert, respectively. The label and prediction probability of each O_Z_ channel signal sample are titled.

Extensive experiments have found that most drowsy samples typically contain a high ratio of theta and delta waves, e.g., as shown in Figure 6A, or alpha waves, e.g., as shown in Figure 6B. From the visualization of amplitude fluctuation and relative power displayed in Figure 6A, we have discovered that both methods have captured several episodes, which contain rhythmic bursts of slow waves in the theta-delta band, which is identified as strong evidence for drowsy classification. Actually, these bursts located in the theta-delta band are closely associated with drowsiness (Britton et al., 2016). From the second sample displayed in Figure 6B, we found that the EEG signal in the alpha band has a stronger amplitude and higher relative power, characterized by the narrow frequency peaks. Compared to CompactCNN, our method has captured more regions of spindle-like structures in the alpha band from the central part of the input signal, recognized as strong evidence of drowsiness. It is well known that the captured alpha spindles in EEG signals have been demonstrated to be a strong indicator for recognizing fatigue driving (Simon et al., 2011). Compared with CompactCNN in Figure 6, we can discover that our method have learned more discriminative effective features such as the slow theta-delta waves and alpha waves, to achieve higher classification accuracy.

It can be seen that most alert samples typically contain a high ratio of beta waves, e.g., as shown in Figure 7A, or a high ratio of delta waves, e.g., as shown in Figure 7B. From Figure 7A, we can discover that both methods have captured some short EEG episodes containing lots of high-frequency beta waves as strong evidence for alertness. Notably, these identified beta waves were typically linked to active, busy, attention, or even electromyography (EMG) activities, which is known as the signals during wakeful state (Baumeister et al., 2008; Goncharova et al., 2003). Compared to CompactCNN, our method has not identified the pattern of one high amplitude peak wave that may be caused by eye blinking or eye movement, as the evidence of alertness. This suggests that our method is not only more resistant to the interference caused by the artifacts in the EEG signals, but it also achieves the superior classification result.

From Figure 7B, we can discover that both methods have captured these large-voltage and low-frequency waves (delta band) as strong evidence for alertness. With these discriminative invariant features found, both two methods have achieved high likelihoods. Due to the fact that delta waves are dominant during the deep sleep phase, they are more likely caused by sensor drifts or subject movements during wakeful state. In fact, these typical patterns in EEG signals including EMG and movements are the strongest indicators for wakefulness (Britton et al., 2016). The visualization results prove that our method achieves higher classification accuracy with neurophysiologically reliable patterns found in single-channel EEG signals.

## 4 Discussion and future work

### 4.1 Analysis of confusion matrixes

**Figure 8:**
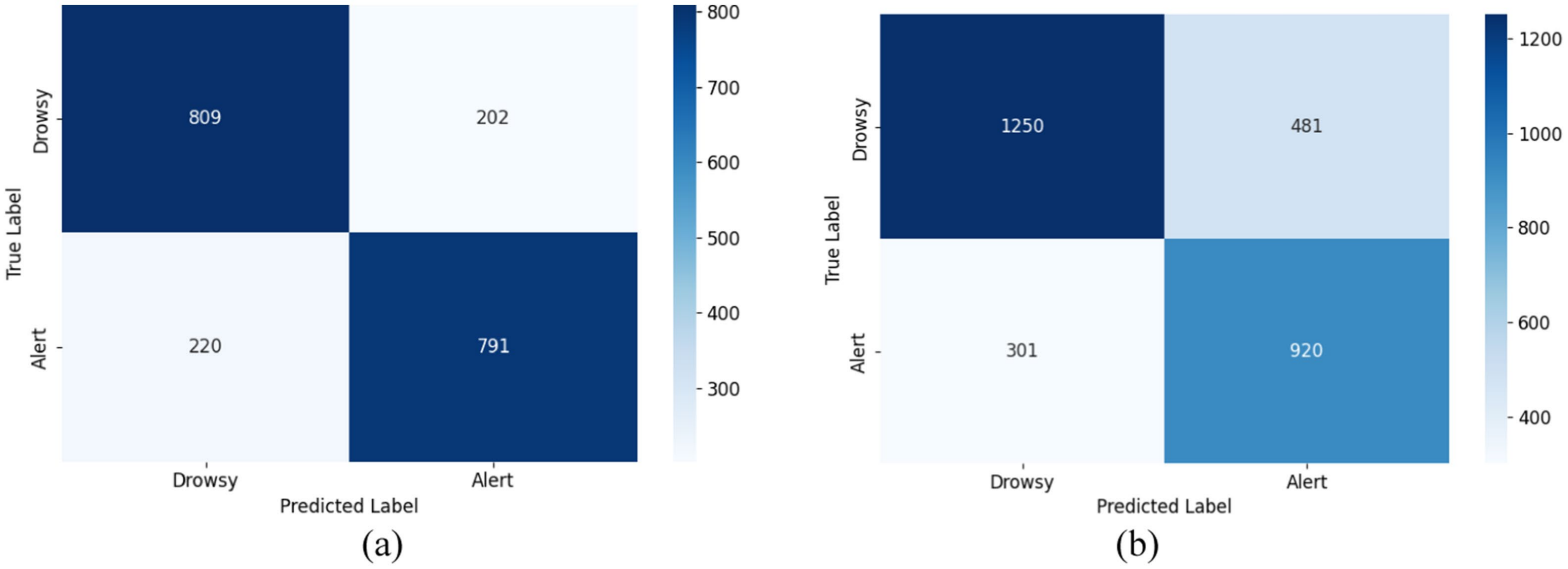
Confounder matrix for two types of data. **(A)** Confusion matrix on the balanced dataset. **(B)** Confusion matrix on the unbalanced dataset.

The confusion matrixes of the proposed ID3RSNet are shown in Figure 8. These matrixes were generated by summing the scores for each subject when serving as test data. In the case of the balanced dataset, Figure 8A shows that the model recognized alert and drowsy states with similar results, correctly predicting approximately 80% of each category. This indicates that the model has a relatively balanced ability to discriminate between different categories. In addition, Figure 8B reveals that the proposed method performed slightly better in recognizing the alert state compared to the drowsy state on the unbalanced dataset since there are more data with drowsy label than the alert. Furthermore, it can be also found that the achieved classification results of the proposed method tested on unbalanced dataset are inferior to the results tested on the balanced dataset, since the unbalanced dataset has 930 more unseen test EEG samples than the balanced dataset.

### 4.2 Analysis of computational complexity

To verify the computational efficiency of the proposed ID3RSNet, we evaluate the testing time for each subject, the FLOPs and the number of parameters on the unbalanced dataset, and the mean training time for each subject on the balanced dataset for different lightweight deep learning methods. All experiments used the same platform with the same hardware configuration and the same software configuration, which are described in Section 3.2. Table 4 indicates that, comparing to the state-of-the-art lightweight methods, the proposed method achieves optimal performance with no significant difference in computational complexity. Even though the proposed model takes a little longer testing time for each subject than other baselines due to the extensive computation of weighting freezing (WF), this speed is still quite fast for driver drowsiness detection.

**Table 4:** Computation time, flops, and parameters with different lightweight deep learning methods.

Methods	ShallowConvNet	CNN-LSTM	CompactCNN	ID3RSNet
Training time (ms)	7.5	5.7	2.2	**1.5**
Testing time (ms)	0.5	0.7	**0.3**	5.2
Flops (M)	50.5	39.4	**32.9**	44.0
Parameters (K)	4.3	2.5	**2.2**	5.6

Bold indicates optimum.

### 4.3 Analysis of different types of thresholds

There are two types of thresholds in residual shrinkage building unit (RSBU): channel-shared (CS) thresholds and channel-wise (CW) thresholds. The RSBU-CW differs from the RSBU-CS by applying an individual threshold to each feature map channel. As shown in Table 5, the results demonstrate the effectiveness of channel-wise thresholds for each channel of the extracted feature map, which is adopted in our proposed ID3RSNet. This also indicates that the RSBU-CW is more effective for eliminating redundant information and automatically selecting important features comparing to the RSBU-CW.

**Table 5:** Classification performance tested on balanced and unbalanced dataset with the methods of two thresholds.

Methods	Balanced dataset		Unbalanced dataset	
	Accuracy	F1-score	Accuracy	F1-score
CS	76.80	**77.37**	74.19	71.20
CW	**77.16**	77.17	**74.72**	**71.27**

Bold indicates optimum.

### 4.4 Considering federated transfer learning in future work

In this study, we explore a promising research topic that an inherently interpretable residual shrinkage network (ID3RSNet) is developed to improve classification performance with interpretable evidence for driver drowsiness detection. To further generalize well to unseen subjects in real-world scenarios, we must try to make further exploration to design transfer learning including unsupervised domain adaptation (UDA) methods, which have the potential in mitigating domain discrepancies among different subjects (Gao et al., 2023a). It is important to note that EEG data contain rich privacy information from each individual, posing a potential risk for privacy leakage when sharing personal source data for training. Federated learning (Rao et al., 2024) that can jointly deploy the deep learning model in the edge devices may address the problem of protecting data privacy and security. In the future, we will focus on the optimized solution of federated transfer learning for driver drowsiness detection with single-channel EEG. With private data for training provided by different edge devices and aligned feature distribution, this solution will further improve generalization performance for privacy-preserving drowsiness detection of source subjects.

## 5 Conclusion

In this study, we propose a novel interpretable residual shrinkage network (ID3RSNet) for cross-subject driver drowsiness detection with single-channel EEG. Soft thresholding and attention mechanisms integrated into the residual shrinkage network are applied to automatically enhance the representation ability of important features. In addition, both the GAP layer and the WF regularization approach are utilized to further improve classification performance. With the inherently interpretable network structure designed, we propose an EEG-based class activation map (ECAM) interpretable method to visualize discriminable common patterns of single-channel EEG signals that contribute significantly to classification. Extensive experimental results indicate that our interpretable model with neurophysiologically reliable evidences, e.g., alpha spindles and theta-delta bursts, achieves the current state-of-the-art performance. Moreover, this study also provides insight into the development of portable single-channel EEG devices with interpretable neural networks for driver drowsiness detection in real-life scenarios.

## Data Availability

The original contributions presented in the study are included in the article/supplementary material, further inquiries can be directed to the corresponding author.
